# Divergent bacterial landscapes: unraveling geographically driven microbiomes in Atlantic cod

**DOI:** 10.1038/s41598-024-56616-y

**Published:** 2024-03-13

**Authors:** Fanny Fronton, Richard Villemur, Dominique Robert, Yves St-Pierre

**Affiliations:** 1 INRS-Center Armand-Frappier Santé Technologie, 531 Boul. des Prairies, Laval, QC H7V 1B7 Canada; 2 Institut des Sciences de la Mer, Université du Québec à Rimouski, 310, allée des Ursulines, C.P. 3300, Rimouski, QC G5L 3A1 Canada

**Keywords:** Atlantic cod, Dysbiosis, Circulating microbiome, 16S rRNA gene, Gulf of St. Lawrence, *Nitrobacter*, *Sediminibacterium*, Metagenome, Microbial ecology, Ocean sciences, Marine biology, Microbiology

## Abstract

Establishing microbiome signatures is now recognized as a critical step toward identifying genetic and environmental factors shaping animal-associated microbiomes and informing the health status of a given host. In the present work, we prospectively collected 63 blood samples of the Atlantic cod population of the Southern Gulf of Saint Lawrence (GSL) and characterized their 16S rRNA circulating microbiome signature. Our results revealed that the blood microbiome signature was dominated at the phylum level by *Proteobacteria*, *Bacteroidetes*, *Acidobacteria and Actinobacteria*, a typical signature for fish populations inhabiting the GSL and other marine ecosystems. At the genus level, however, we identified two distinct cod groups. While the microbiome signature of the first group was dominated by *Pseudoalteromonas*, a genus we previously found in the microbiome signature of Greenland and Atlantic halibut populations of the GSL, the second group had a microbiome signature dominated by *Nitrobacter* and *Sediminibacterium* (approximately 75% of the circulating microbiome). Cods harboring a *Nitrobacter/Sediminibacterium*-rich microbiome signature were localized in the most southern part of the GSL, just along the northern coast of Cape Breton Island. Atlantic cod microbiome signatures did not correlate with the weight, length, relative condition, depth, temperature, sex, and salinity, as previously observed in the halibut populations. Our study provides, for the first time, a unique snapshot of the circulating microbiome signature of Atlantic cod populations and the potential existence of dysbiotic signatures associated with the geographical distribution of the population, probably linked with the presence of nitrite in the environment.

In recent years, the marine ecosystems of the Northwest Atlantic have been warming rapidly and are undergoing a rapid transition, jeopardizing the persistence of some once predominant species (e.g.,^1^). The Atlantic cod (*Gadus morhua*), a widely distributed groundfish of high economic and cultural importance throughout the region, has remained at relatively low abundance following a massive collapse in the 1990s due to overfishing during a period of low productivity^2^. Moreover, some cod populations face further declines even without fishing pressure, which is the case for those distributed in the southern Gulf of St. Lawrence (SGSL), which is considered vulnerable to extinction^3^. Recent data have revealed an extremely high natural mortality rate of 55% in adult SGSL cod, a significant departure from the expected 20%^3^. Although increased predation by the grey seal (*Halichoerus grypus*) likely constitutes the primary factor driving this decline^4^, the health status of Atlantic cod may also be impacted by changes in the environment, leading to concurrent changes in its physiology, immune response and susceptibility to infectious diseases^5–8^. Several previous studies have indeed reported the role of environmental factors in limiting Atlantic cod conditions and productivity (e.g.,^9,10^).

To better understand the role of the environment in Atlantic cod population dynamics, it becomes imperative to set up programs for monitoring the health status of Atlantic cod populations and to develop new sensitive and predictive biomarkers compatible with the sampling logistics of the current monitoring programs. Currently, individual health assessments of wild fish populations remain relatively scarce, mainly relying on calculating the condition factor K with individual length and weight as a measure of “plumpness”^11^. Even though this method is fast and easy to measure, its sensitivity and predictive value remain relatively low^12^. Additional methods include data obtained from tissue biopsies, which, unfortunately, are costly and logistically complicated^13,14^.

In humans, liquid biopsy-based biomarkers have emerged as critical tools for the follow-up of the health status of patients and for detecting signs of the onset of diseases at their earliest stage. Initially developed in oncology to detect mutations in circulating cell-free DNA in the blood of patients with cancer, liquid biopsy-based biomarkers have been extended to the analysis of other omics, including the microbiome. The importance of establishing the host microbiome signature has gained interest in recent years, notably since landmark studies revealed that it is possible to identify specific microbiome signatures that predict the outcome of treating cancer patients with immunotherapy^15,16^. This approach is now commonly used to compare hosts afflicted with various types of disorders to healthy populations, allowing clinicians to establish a diagnosis, predict future onset of pathologies, and even predict treatment success probability as a function of physical fitness. The approach gained further clinical interest when applied to the blood, leading to the concept of the circulating bacterial microbiome. In this case, the focus is on analyzing bacterial nucleic acids found in the host's blood, allowing the detection of potential pathogens and dysbiosis induced by environmental changes. Dysbiosis, defined here as a change in the abundance or diversity of some groups of microorganisms, is very sensitive to various forms of environmental stress, such as the response to elevated temperatures^17^. Recent studies have applied this concept to marine species, including invertebrates and fish populations^18,19^. For example, using a single drop of blood fixed on cellulose paper, we recently reported that the circulating microbiome signature of wild halibut populations is sensitive to physiological and environmental factors^20^.

In the present work, we prospectively collected blood samples from the SGSL Atlantic cod population in September 2021 and studied the circulating microbiome signature by sequencing the 16S ribosomal RNA (rRNA) gene sequence. This study aimed to characterize the circulating microbiome of wild fish and correlate it with physiological and environmental data, giving us more information on their general health status. Our results suggest the existence of dysbiotic signatures associated with the geographical distribution of the cod population.

## Material & methods

### Sampling

Blood samples from individual Atlantic cod (n = 63; 15–57 cm) were collected between September 10 and September 26, 2021, during the second leg of the annual Fisheries and Oceans Canada (DFO) bottom trawl survey in the southern sector of the SGSL, Canada (Table 1**)**. Blood samples were taken from the caudal vein immediately upon trawl retrieval, and overall, liquid biopsies were performed at 21 sites for at least one individual. The number of liquid biopsies carried out per station (ranging from 1 to 6) was opportunistic and depended on the presence of Atlantic cod and workload at a given site. Blood samples were collected with a heparin-coated 3 mL sterile syringe and a 22G needle and immediately stored on an FTA™ card (Sigma‒Aldrich®, Oakville, ON, Canada) to minimize contamination. Samples were allowed to air dry in a separate (dry) lab and stored in a plastic bag with a desiccant, as described by Caza et al.^18^. Drops of blood were collected and immediately stored on an FTA™ card (Sigma‒Aldrich®, Oakville, ON, Canada). Samples were allowed to air dry and stored in a plastic bag with a desiccant, as described by Caza et al.^18^. The sex of each sampled individual was determined by visual identification of the gonads following the dissection of specimens by the DFO science crew. Scanmar® hydroacoustic sensors attached to the trawl and a conductivity, temperature, and depth (CTD) probe were used to record environmental characteristics. The care and use of field-sampled animals complied with the Government of Canada animal welfare laws, guidelines, and policies approved by Fisheries and Oceans Canada. All methods are reported in accordance with ARRIVE guidelines (https://arriveguidelines.org/).

**Table 1:** Summary of the fish samples used for cmDNA analysis.

	n	Length (cm) mean ± SE	Weigth (g) mean ± SE
Male	21	40.38 ± 1.65	609.12 ± 88.01
Female	40	37.90 ± 1.44	550.33 ± 66.68
Unknown	2	25.00 ± 14.14	165.50 ± 197.28
Total	63	38.32 ± 1.13	557.71 ± 51.68

### DNA extraction, amplification and sequencing

All DNA extraction and purification procedures were conducted in a clean room where pressure, temperature, and humidity were controlled to minimize contamination. Individual discs were cut from the FTA™ cards using a sterile 5.0 mm single round hole punch, and total DNA was isolated using the QIAamp DNA Investigator Kit (Qiagen, Toronto, ON, Canada) according to the manufacturer’s protocol. DNA was quantified in duplicate using a Quant-iT™ PicoGreen® dsDNA detection kit (Molecular Probes, Eugene OR, USA). Amplification of the V3–V4 region of the 16S rRNA gene and 16S rRNA gene amplicon sequencing for all DNA samples were performed at Centre d'Expertise et de Services Génome Québec (Montréal, QC, Canada) using the universal primers 341F (5′-CCTACGGGNGGCWGCAG-3′) and 805R (5′-GACTACHVGGGTATCTAATCC-3′). Sequence libraries were prepared by Genome Quebec with the TruSeq® DNA Library Prep Kit (Illumina, San Diego, CA, USA) and quantified using the KAPA Library Quantification Kit for Illumina platforms (Kapa Biosystems). Paired-end sequences were generated on a MiSeq platform PE300 (Illumina Corporation, San Diego, CA, USA) with the MiSeq Reagent Kit v3 600 cycles (Illumina, San Diego, CA, USA). Raw data files are publicly available in the NCBI Sequence Read Archive (PRJNA1015160).

### 16S rRNA gene data processing and data analysis

Illumina sequence data (FASTQ files) were trimmed using *Cutadapt* (version 2.8). The 16S rRNA gene (V3–V4) amplicon sequence variants (ASVs) were generated with the DADA2 pipeline (version 1.16.0^21^;) and subsequently within the R environment (R version 4.1.1, Team (2022)). The RDP 16 database was used for the ASV assignment. The software packages *phyloseq* (1.36.0), *microbiomeSeq* (0.1), *microbiomeMarker* (0.99.0) and *vegan* (2.5.7) were used to characterize the microbial communities^22–25^. The maps were created with the packages *ggplot2* (3.3.6) and *rnaturalearth* (0.1.0)^26^. An ASV was considered part of the core microbiome if it had a minimum prevalence (rate of presence in the group of samples) of 70%, with a detection threshold of 0.01% relative abundance, as described by Palanisamy and colleagues^27^. A similar decision tree was applied for the core genera (abundance of ASVs of the same genus summed up) but with 90% prevalence and a detection threshold of 0.01%, as described by Fronton and colleagues^20^.

Bacterial taxonomic α-diversity (intrasample) was estimated using the richness and the Shannon and Simpson indices implemented in the R package *microbiome* (1.14.0). Variations in bacterial α-diversity and taxon abundances between the two populations were assessed using either the Kruskal‒Wallis test or the Wilcoxon–Mann‒Whitney test since none of the variables had a normal distribution. α-Diversity was also calculated among maturity stages according to the length at maturity (L50), which is estimated at 40 cm for this species^3^, the sex and the relative condition factor K^28^. The Kruskal‒Wallis test was followed by a pairwise Wilcoxon–Mann‒Whitney test if the *p* value (*p*) was significant (*p* < 0.05). The β-diversity (intersample) was estimated using phylogenetic weighted UNIFRAC dissimilarities assessed by principal coordinates analysis (PCoA). Differences in community composition were tested using permutational multivariate analysis of variance (PERMANOVA) for weighted UNIFRAC indices with 999 permutations, as implemented in the R *vegan* package (2.5.7) or the *pairwise Adonis* package (0.4). A redundancy analysis (RDA) was performed on the standardized (Hellinger transformation) number of reads for each ASV in each matrix and the environmental and physiological variable matrix^29^. The collinearity between variables was validated with a Pearson correlation, and weight and length were the only variables correlated. Differences were considered statistically significant at *p* < 0.05. Data analyses were performed in R studio (v4.0.5).

### Condition K factor

The relative condition K factor, a broad health index for fish, was calculated based on the length and weight of each individual^28,30^. A linear regression was performed between log_10_ (weight) and log_10_ (length) as follows^31^:

$${log}_{10}\left(W\right)={log}_{10}\left(a\right)+{b*log}_{10}(L)$$

Where W is the weight, L is the length, and a and b are constant coefficients.

The coefficients a and b were calculated and used to estimate the expected weight W_e_ of each individual based on their length with the following equation:

$${W}_{e}=a{L}^{b}$$

Finally, the K_rel_ of Le Cren was calculated as follows:

$${K}_{rel}= \frac{W}{{W}_{e}}$$

The comparison between the individual’s actual and expected weight gives us the fish's plumpness, a glimpse of its health status. If an individual is skinnier than others of the same length within the same species, it is considered to undergo stress, and K_rel_ will be under 1. In contrast, a plump fish is associated with advantageous environmental influence, and K_rel_ will be over 1^11^.

## Results

### Preliminary characterization of the circulating microbiome

The circulating microbiome signatures were determined by sequencing the V3–V4 hypervariable regions of the 16S rRNA gene. Approximately 2,800,000 raw reads were retrieved after filtering. The number of sequences per sample ranged between 5154 and 111,505. The mean number of reads per individual was 44,620.43 ± 29,34.44. The number of ASVs per sample curve confirmed that the sequencing depth was sufficient to plateau the number of ASVs (Fig. S1). A total of 920 unique ASVs were obtained, with 47.37 ± 1.58 ASVs on average per individual.

**Figure 1:**
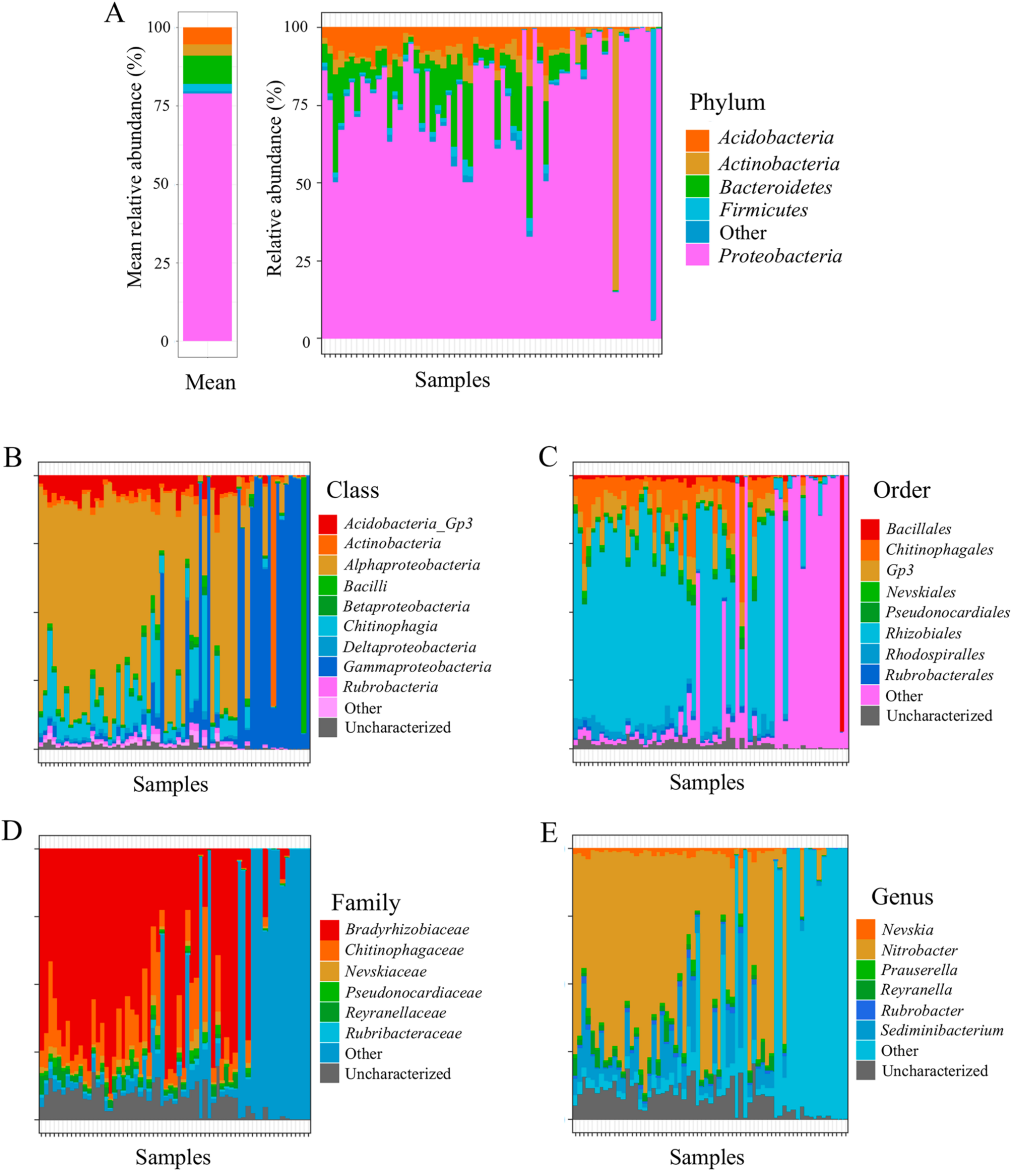
Relative abundance of the main taxa in the circulating microbiome of Atlantic cod. (**A**) Mean relative abundance at the phylum level. (**B**) Relative abundance of individual taxa at the class level. (**C**) Relative abundance of individual taxa at the order level. (**D**) Relative abundance of individual taxa at the family level. (**E**) Relative abundance of individual taxa at the genus level. Data was obtained from a sample size of n = 63. The prevalence of taxa analyzed was 80%, with a detection threshold of 0.01%.

Overall, in the 63 samples we analyzed, we identified 920 ASVs at different taxonomic levels, including 18 phyla, 32 classes, 63 orders, 101 families, and 168 genera. At the phylum level, the blood microbiome signature was dominated by *Proteobacteria*, *Bacteroidetes*, *Acidobacteria and Actinobacteria,* representing an average of 96.89% of the microbiome. The prevalence of *Proteobacteria* was particularly dominant, with some samples showing almost 100% *Proteobacteria* in their microbiome (Fig. 1A). However, our analyses at the class, order and family levels revealed two very different signature groups. At the class level, the first group was primarily dominated by the presence of *Alphaproteobacteria,* while the second group was characterized by the dominance of *Gammaproteobacteria* (Fig. 1B). This second group also showed substantial heterogeneity at the order, family and gene levels, a notable difference from the first group dominated by *Rhizobiale*s and *Bradyrhizobiaceae* (Fig. 1C,D).

### Identification of two groups with distinct microbiome signatures.

**Figure 2:**
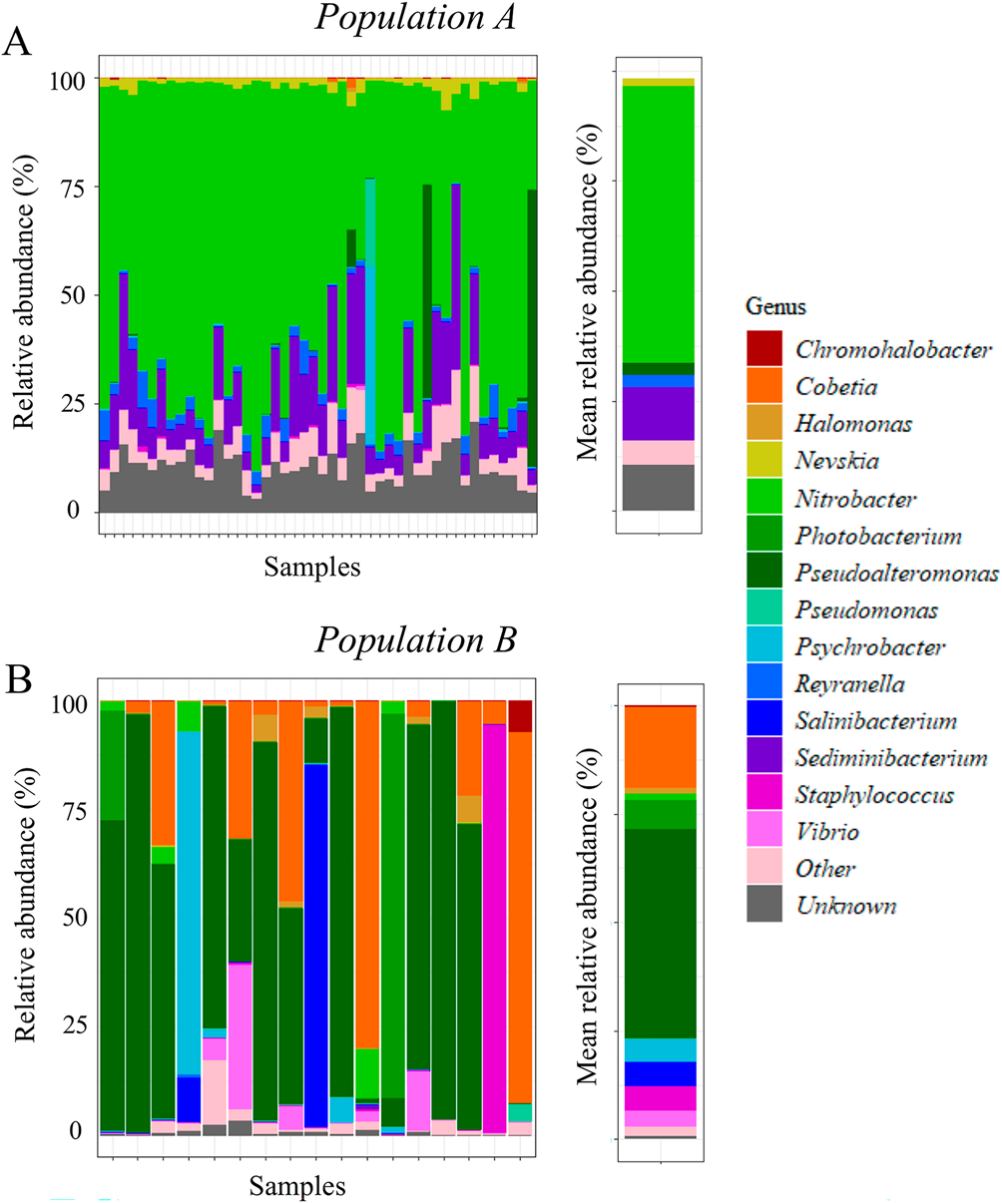
Relative abundance of the main genera in the circulating microbiome of Atlantic cod. (**A**) Main genera found in the circulating microbiome samples of Population A, with mean values. Population A consisted of 46 individuals, where the relative abundance of core microbiome taxa was > 30%. Prevalence of taxa analyzed was 1%, with a detection threshold set at 5%. (B) Main genera found in the circulating microbiome samples of Population B, with mean values. Population B consisted of 17 individuals, where the relative abundance of core microbiome taxa was < 30%. Prevalence of taxa analyzed was 1%, with a detection threshold set at 5%.

Next, we investigated the contrasting microbiome signatures in detail at the genus level (Fig. 1E). We found that the first group (Group A, n = 46) had a relatively homogeneous microbiome signature dominated by the *Nitrobacter* and *Sediminibacterium* genera, both of which are involved in the nitrogen cycle^32^. On average, *Nitrobacter* accounted for 63.06 ± 2.82%, and *Sediminibacterium* accounted for 12.27 ± 1.31% of the circulating microbiome of group A (Fig. 2A). Group B (n = 17) was defined as every individual with a low (< 30%) abundance of *Nitrobacter* and *Sediminibacterium,* the two genera part of the core microbiome (Fig. 2B and S2). This population was more heterogeneous and dominated by *Pseudoalteromonas* and *Cobetia*, which accounted for 48.03 ± 9.58% and 18.69 ± 7.01% of the circulating microbiome, respectively.

### The α and β-diversity of the Atlantic cod circulating microbiome.

**Figure 3:**
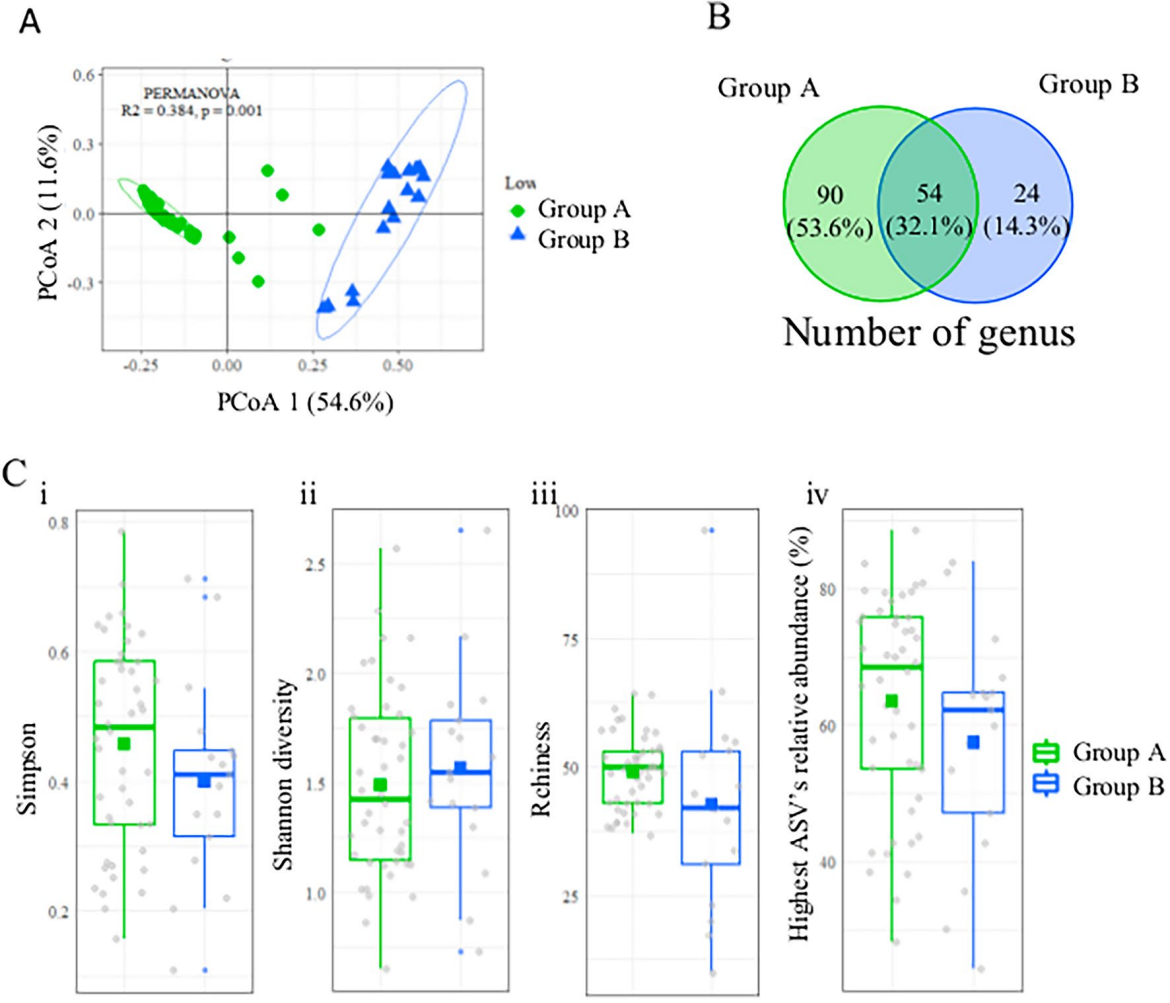
Diversity indices comparison of the circulating microbiome in two groups of Atlantic cod. (**A**) Principal Coordinates Analysis (PCoA) plot based on the weighted UniFrac distance, illustrating the differentiation between the two populations of cod circulating microbiomes. (**B**) Venn diagram depicting the number of genera observed in each population. (**C**) α-Diversity indices for both cod microbiome groups, represented by solid squares denoting the mean values. Group A consisted of individuals with a relative abundance of core microbiome > 30%, while Group B consisted of individuals with a relative abundance of core microbiome < 30%. The indices analyzed include (i) Simpson's index, (ii) Shannon's diversity index, (iii) Number of ASVs observed, and (iv) Relative abundance of the most abundant ASV. The sample size was n = 63, with Group A consisting of 46 individuals and Group B consisting of 17 individuals.

**Figure 4:**
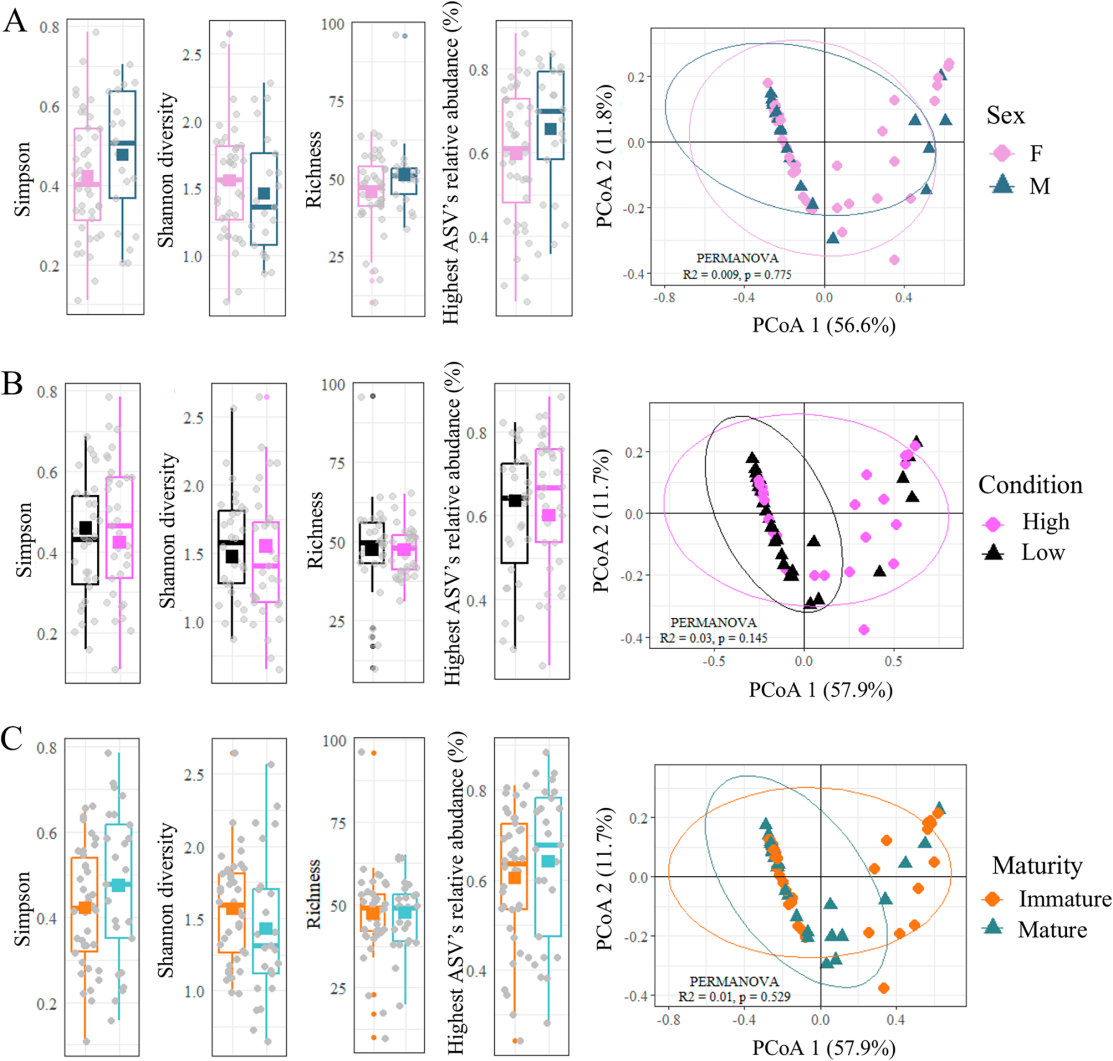
Diversity indices comparison of physiological variables in the circulating microbiome of Atlantic cod. The diversity indices analyzed include Simpson's index, Shannon's diversity index, the number of ASVs observed, and the relative abundance of the most abundant ASV. Mean values are represented by solid squares. Additionally, a Principal Coordinates Analysis (PCoA) based on the weighted UniFrac distance is shown. The PCoA plot visualizes the differentiation in the circulating microbiome based on the physiological variable being compared. (**A**) Comparison between sexes: The sample size was n = 61, with 21 individuals classified as males (M) and 40 individuals classified as females (F). (**B**) Comparison between condition classes: The sample size was n = 63, with 30 individuals classified as having high condition and 33 individuals classified as having low condition. (**C**) Comparison between maturity classes: The sample size was n = 63, with 38 individuals classified as immature and 25 individuals classified as mature.

To further investigate the difference in the microbial composition between the two groups, we carried out a principal coordinate analysis (PCoA). Studies of the community composition by a PERMANOVA revealed a significantly different clustering (*p* = 0.001), with the separation in two groups explaining 38.4% of the variations (R^2^ = 0.382) (Fig. 3A)". The Venn diagram of the number of genera in each population showed a more diverse microbiome in group A, with 90 unique genera against 24 in group B. However, both groups shared 54 genera (32.1% of all the genera) (Fig. 3B). When we measured the α-diversity indices of the microbiomes of both populations, including the Shannon diversity index, Simpson’s index, richness, and relative abundance of the most abundant ASV, we found no significant difference (Fig. 3C). Otherwise, there was no difference in the α or the β-diversity when considering sex, relative condition, or maturity classes (Fig. 4).

### Geographical distribution of the cod population with distinct blood microbiomes.

**Figure 5:**
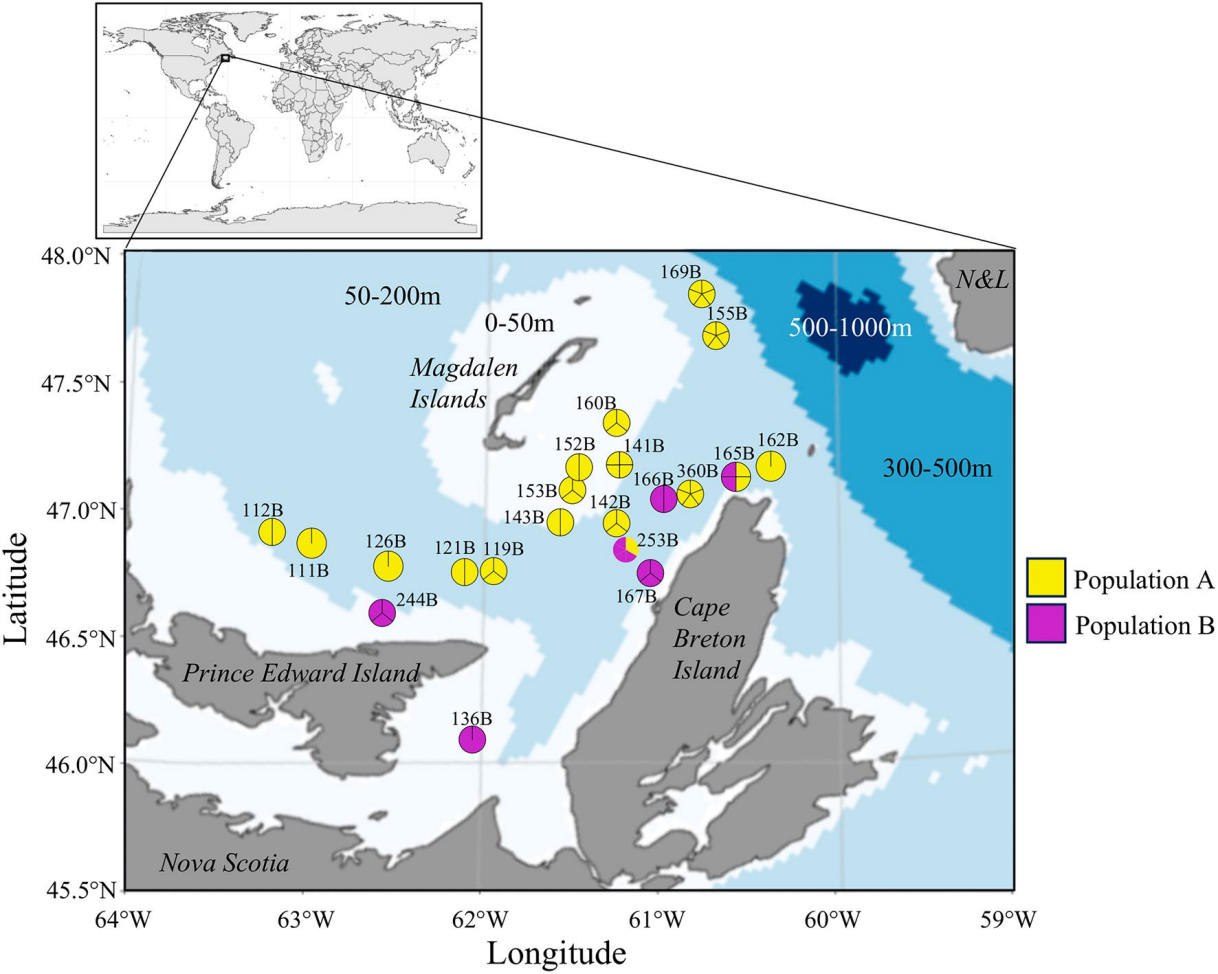
Map indicating the 21 sampling sites. The seafloor depth is labeled on the map, and the number of cod individuals in Group A (yellow) or Group B (purple) is displayed at each site. The lines within the circles denote the number of samples collected at each specific station. N&L represents Newfoundland & Labrador. The figure was generated using ggplot2 *sf* (1.0–13), *rnaturalearthdata* (0.1.0), *sf*(1.0–13), and *rnaturalearthdata* (0.1.0), *ggOceanMaps* and *ggOceanMapsData* (1.3.4), and the *scatterpie* package (0.1.8) in R (version 3.4.0) and Inkscape.

Next, we examined which factors could explain the presence of the two distinct signatures in the blood microbiome. A redundancy analysis (RDA) was thus performed on the normalized number of reads for each ASV in each matrix and the environmental and physiological variable matrix. An ANOVA-like permutation test for RDA revealed that weight, length, relative condition index (K_rel_), depth, temperature, sex, and salinity did not significantly influence the Atlantic cod circulating microbiome (Table 2). We did find, however, a statistically significant correlation between the blood microbiome signature and sampling sites (*p* < 0.001). We found that individuals with a *Nitrobacter*-rich microbiome (group A) were located near Cape Breton Island, whereas group B was closer to the Magdalen Islands (Fig. 5). Of the 21 sampling sites, only one, 165B, contained a mix of populations A and B.

**Table 2:** Results of the ANOVA-like permutation test on the RDA made on the number of reads by ASV (Hellinger transformation) with physiological and environmental variables.

Variable	Degree of freedom	Variance	*p* values	
Length	1	0.007	0.299	(ns)
Weight	1	0.014	0.105	(ns)
Depth	1	0.011	0.149	(ns)
Temperature	1	0.006	0.452	(ns)
Salinity K_rel_ Sex Station Residuals	1 1 2 13 28	0.015 0.003 0.030 0.205 0.188	0.065 0.788 0.053 0.001	(ns) (ns) (ns) (***)

## Discussion

The present work aimed to assess the circulating signature and diversity of the circulating blood microbiome of the Southern Gulf of St. Lawrence Atlantic cod, a population that faces extinction following decades of continuous decline. At the phylum level, the blood microbiome signature was dominated by *Proteobacteria*, *Bacteroidetes*, *Acidobacteria and Actinobacteria.* Apart from a few minor differences, particularly concerning the lower prevalence of *Firmicutes*, this taxonomic structure at the phylum level was similar to that recently described in the circulating microbiome of the Greenland halibut (*Reinhardtius hippoglossoides*) and Atlantic halibut (*Hippoglossus hippoglossus*) in the Gulf of St. Lawrence^20^. However, we could distinguish two populations with distinct blood microbiome signatures at the lower ranks. The presence of Alphaproteobacteria primarily dominated the first signature. At the same time, the second showed the dominance of *Gammaproteobacteria* and a very heterogeneous signature at the order and family levels, a notable difference from the first group dominated by *Rhizobiale*s and *Bradyrhizobiaceae.* Interestingly, the second group had a signature dominated by the *Nitrobacter* and *Sediminibacterium* genera, which are involved in the nitrogen cycle^32^. Finally, we found that the taxonomic and phylogenetic structures of the bacterial community were restricted to specific regions of the SGSL, suggesting that the environment directly impacts the circulating microbiome. The data revealed exciting clinical prospects for using a blood microbiome genetic signature for detecting dysbiosis, risk stratification, and disease surveillance of the cod population in response to environmental changes.

In medicine, the characterization of the peripheral blood-derived microbiome signature, defined as blood microbial DNA, is increasingly used by clinicians to assess an individual's health status, detect dysbiosis and potential pathogens, or as a biomarker to inform disease severity and progression^33–37^. This concept is also gaining momentum in ecology as the circulating microbiome of dogs, bovine, wild birds and wild fish populations were recently studied, showing that the genetic structure of the blood microbiome, just as in humans, is modulated by genetic and spatiotemporal factors, as well as disease conditions^20,38–41^. In the present study, the observed shift toward the dominance of *Nitrobacter* and *Sediminibacterium* suggests that environmental factors severely impact the blood microbiome signature. The presence of *Sediminibacterium* is not uncommon in aquatic species^42–44^. In trout, the presence of this genus is sensitive to seasonal changes^45^. Its presence is also not uncommon in dysbiotic microbiome profiles. In humans, for example, its presence is associated with lung cancer diagnosis and is in higher abundance in the circulating microbiome of type 2 diabetes mellitus patients^46,47^. The presence of *Nitrobacter* is also not uncommon in dysbiotic microbiomes. The *Nitrobacter* genus plays a role in the nitrogen cycle, as it can oxidize nitrite (NO_2_^−^) to nitrate (NO_3_^−^). To our knowledge, however, this dominance of *Nitrobacter* has not been reported in wild fish populations in the past, although bacteria associated with the metabolism of nitro compounds have been found, albeit at lower levels, in the blood microbiome, gut or skin microbiome of various animals, including bovine and fish^40,48–50^. Experimentally, however, exposure of goldfish to nitrite has been shown to induce a shift in the gill, nose and skin microbiome toward bacterial communities involved in the nitrogen cycle and the disappearance of taxa generally found in the microbiome^51^. Usually, nitrite, an intermediate stage in the nitrogen balance, should not be detectable in a stable environment as it is rapidly transformed into NO_3_^−^ by biodegrading bacteria such as *Nitrobacter* or by chemical reaction in the water. It is also quickly consumed by algae at the surface. Yet, high surface nitrite/nitrate concentrations are not uncommon in marine coastal ecosystems, as it is commonly released in seawater because of agricultural activity^52–54^. Whether this dysbiotic microbiome signature is associated with health issues in cod will require future investigation.

The presence of *Nitrobacter* and *Sediminibacterium* in population A contrasted with the microbiome profile of population B, where we found DNA derived from *Pseudoalteromonas,* a genus of marine bacteria commonly found in marine species, such as sponges, shellfish, macroalgae and fish, including wild fish populations of the Greenland halibut and Atlantic halibut of the Gulf of St. Lawrence^20,55–58^. Considered a mutualistic bacterium that plays a vital role in the fitness and survival of its host, this genus is known to adapt well to cold environments. It can synthesize bioactive compounds with strong antibacterial and antitumor properties^55,59, 60^.

Our study revealed the existence of two distinct microbiome signatures in cod populations. We found, however, no significant difference in the α-diversity of both microbiomes. Differences in sex, relative condition or maturity classes were neither associated with a specific signature. Similar conclusions were drawn when we looked for temperature, salinity, or depth differences. Our analysis showed, however, that individuals with a *Nitrobacter*-rich microbiome (group A) were explicitly found in samples collected near the north coast of Cape Breton Island. Whether this is explained by a specific diet, environmental conditions, or distinct migratory patterns combined with seasonal variations is presently unclear. All these factors have been shown to impact the microbiome of marine fish populations^57,61–66^. Seasonal variations may also explain such variations. For example, during winter, the SGSL contains high concentrations of total nitrate (NO_2_^−^ + NO_3_^−^), which is later consumed in the spring by algal blooms (Fig. S3)^67^. The presence of *Nitrobacter* reflects a high concentration of nitrite, but whether the GSL nitrite composition has been impacted by humans (agricultural and aquacultural waste) or by a natural origin in currents remains unclear. Alternatively, both signatures may reflect distinctive migratory behavior.

While our study did not identify potential pathogens, it is important to acknowledge that the analysis of the 16S rRNA gene may not be optimally suited for pathogen detection in a host. One limitation is its limited resolution to distinguish strains or genetic variants within a species, which can hinder the differentiation of pathogenic strains from non-pathogenic ones or tracking the progression of an infection. Additionally, this approach may lack specificity in certain cases. Similar sequences may be found in non-pathogenic bacteria or even in bacteria from other species, leading to potential false positives or difficulties in accurately identifying the target pathogens. To overcome these challenges, more targeted approaches would be necessary to address this issue. It is also important to note that dysbiosis is not solely about shifts in microbial composition but also reflects alterations in microbial functions. Studying dysbiosis at higher taxonomic levels can help assess the functional potential of the microbiota, such as the presence of specific metabolic pathways or the production of bioactive molecules, which can influence host-microbe interactions and disease outcomes.

It is important to remember that our study is based on DNA sequencing analysis of DNA extracted from blood samples. Bacterial DNA found in a blood sample can have diverse origins, including various niches such as the intestines, skin, gills, and oral cavity, among others. It can exist in different forms, such as dormant bacteria, living bacteria originating from the same environment (autochthonous), living bacteria from external sources (allochthonous), degraded bacteria, or solely bacterial DNA without viable cells. It does not necessarily reflect the presence of bacteria in the blood. Although the presence of bacteria in the blood of healthy individuals remains debatable, this does not hold in diseased individuals where damaged epithelial membrane integrity is impaired, leading to dysbiotic profiles in the blood microbiome^68^. Such changes in the blood microbiome are increasingly used to delineate the onset, progression and treatment of diseases, not only in the case of infectious diseases but also in a variety of other conditions, including cancer, metabolic, neurological and cardiovascular diseases, as well as for detecting behavioral anomalies^33,36, 69, 70^. The use of the blood microbiome in marine biology is only in its infancy, but it offers a new perspective to better assess the health status of wild fish populations.

## Conclusions

In summary, our study provides a snapshot of the circulating microbiome signature in an Atlantic cod population and the existence of clear spatial patterns in dysbiotic signatures. Because this logistically friendly approach is compatible with multi-omics analysis, allowing further measurements of changes at the transcriptomic, epigenomic, metabolomic, and glycomic levels, liquid biopsies hold significant potential for better assessing the impact of climate change on the health of cod populations and other marine resources.

### Supplementary Information

Supplementary Figures.

## Data Availability

The sequence data supporting this study's findings are available on the NCBI website at https://www.ncbi.nlm.nih.gov/bioproject/PRJNA1015160. The full dataset is available at https://figshare.com/s/25f476865d094bec1b8f, and the Rstudio code is available at https://figshare.com/s/dde39f44befc0343559b.

## Abbreviations

GSL: Gulf of St. Lawrence
*G.**morhua*: *Gadus* *morhua*
PCoA: Principal coordinate analysis
ASV: Amplicon sequence variants
PERMANOVA: Multivariate analysis of variance with permutation
RDA: Redundancy analysis
