# Supplementary material for: Divergent bacterial landscapes: unraveling geographically driven microbiomes in Atlantic cod
Source: Sci Rep. 2024 Mar 13;14:6088. doi: 10.1038/s41598-024-56616-y (PMC10938007; doi:10.1038/s41598-024-56616-y)
Supplement: Supplementary file 1 — Supplementary Figures. [file 41598_2024_56616_MOESM1_ESM.pdf]

Divergent bacterial landscapes:

Unraveling geographically driven dual microbiomes in Atlantic cod (*Gadus morhua*) from the southern Gulf of St. Lawrence

Fanny Fronton[1], Richard Villemur[1], Dominique Robert[2], and Yves St-Pierre[1]

1) INRS-Center Armand-Frappier Santé Technologie, 531 Boul. des Prairies, Laval, QC, Canada, H7V 1B7

2) Institut des Sciences de la Mer, Université du Québec à Rimouski, 310, allée des Ursulines, C.P. 3300 Rimouski, QC, Canada, G5L 3A1

Supplementary Material

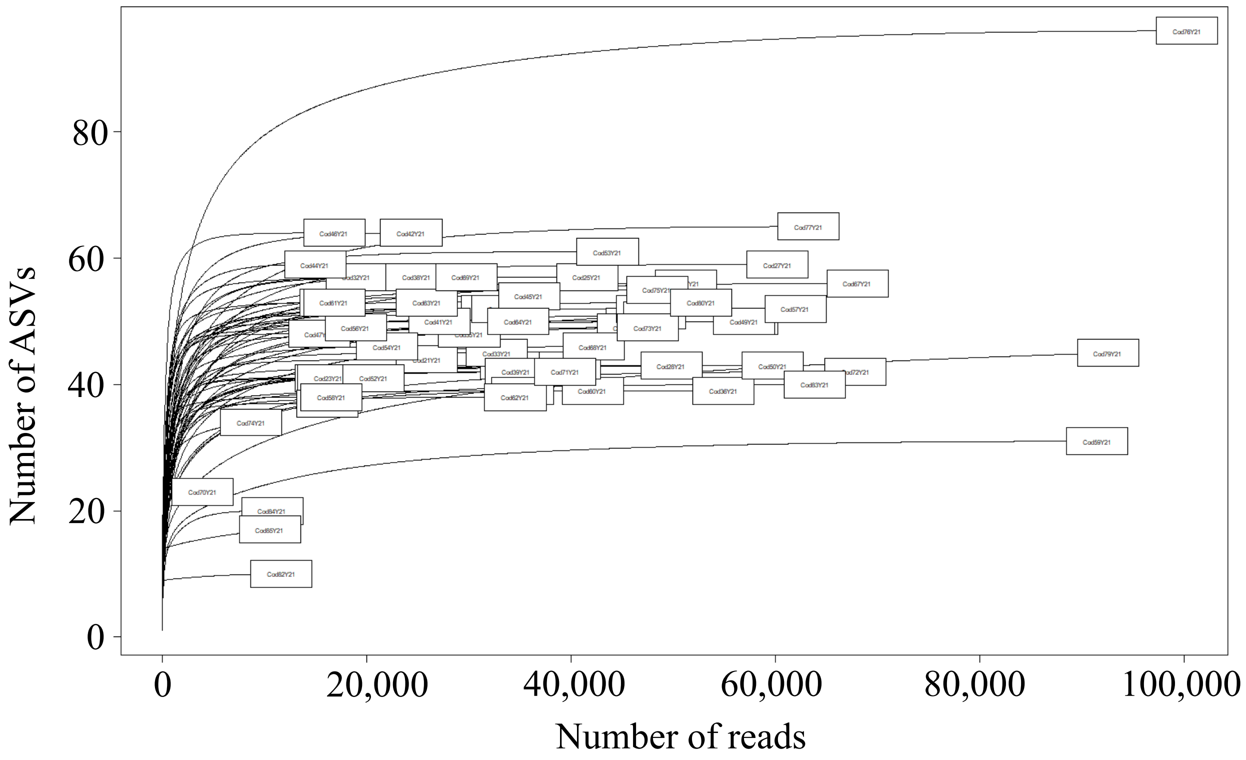

Figure S1: Sequencing depth curves based on the number of reads (sample size) and the number of ASVs for each sample. Each line represents one sample. n=63

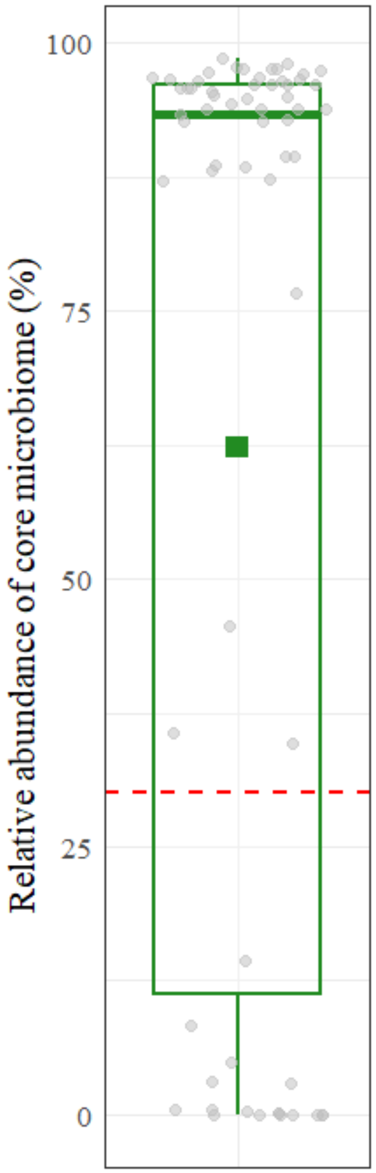

Figure S2: Relative abundance of Atlantic cod's core circulating microbiome. Relative abundance of the core bacteria present the blood microbiome of the Atlantic cods. n = 63. Core microbiome decision: Prevalence = 50%, detection threshold = 0.2%. Red dashed line = 30%. Group A >30%, Group B<30%.

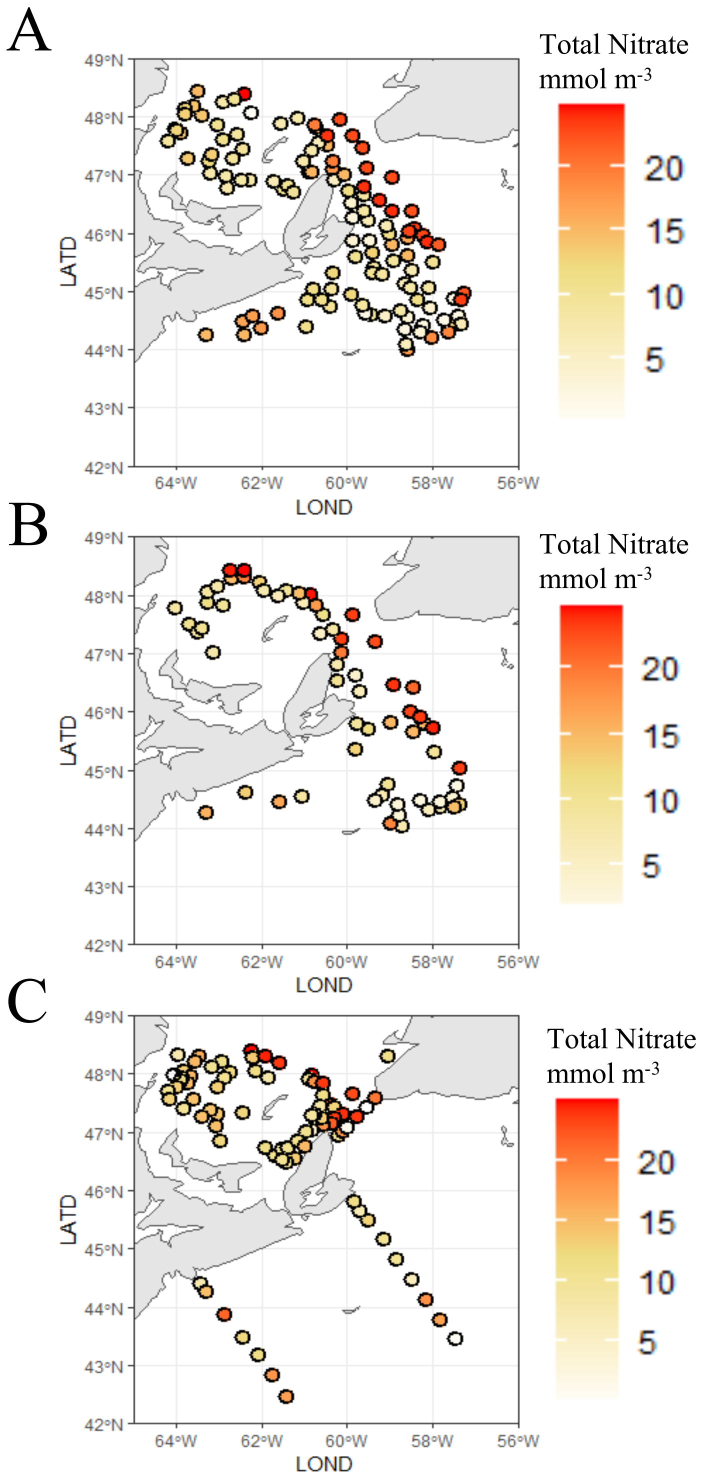

Figure S3: Map of total nitrate (nitrate + nitrite) at the bottom (> 150m depth). (A) Late summer (August to September) (B) Late summer 2020 (C) Late summer 2021. These maps were generated using the MPO data available in Casault *et al*. (DFO Can Sci Advis Sec. Res. Doc. 2023/016, 2023) and Blais *et al*. (Can Tech Rep. Hydrogr. Ocean Sci. 357, 2023) using ggplot2 *sf* (1.0-13), *rnaturalearthdata* (0.1.0), *sf* (1.0-13), and *rnaturalearthdata* (0.1.0), *ggOceanMaps* and *ggOceanMapsData* (1.3.4), in R (version 3.4.0).
